# The professed effect of stigma on community psychiatric nurses in the Greater Accra region of Ghana

**DOI:** 10.1186/s12888-022-04089-6

**Published:** 2022-07-07

**Authors:** Abigail Ansere Buertey

**Affiliations:** 1 Department of Mental Health, School of Nursing and Midwifery, University of Ghana, Legon, Accra, Ghana; 2 School of Nursing, College of Health Sciences, University of Ghana, P.O. Box LG 43, Legon, Accra, Ghana

**Keywords:** Community psychiatric nurses, Communities, Metropolis, Professed effects, Stigmatization

## Abstract

**Background:**

Stigma is a major factor that inhibits Mental Health Nurses work, especially Community Psychiatric Nurses, in terms of productivity. Even though mental health services have improved drastically, because of decentralization of mental health care, a lot more people still have reservations when it comes to mental health nurses particularly Community Psychiatric Nurses. The purpose of the study was to explore the professed effects of stigma on CPNs in the Southern part of Ghana.

**Methods:**

The study was carried out in three district hospitals (Ga South, Ga Central and Okaikoi) all in the Accra Metropolis. The aim of the study was to describe how stigma affects Community Psychiatric Nurses. A qualitative descriptive exploratory design was adopted for the study. The purposive sampling technique was used to recruit participants. Data was saturated with 12 participants, aged between 25 and 40 years. The audio-taped interviews were transcribed verbatim and afterwards analyzed using thematic and content analysis.

**Results:**

The findings gathered from participants revealed that Community Psychiatric Nurses experienced various effects of stigma, such as low productivity, depression, and anger. Most of the participants recounted how stigmatization had affected their work both in the hospital setting and in their communities.

**Conclusion:**

The study showed that Community Psychiatric Nurses carried out their activities with much difficulty, because of their poor image. They stressed the need for recognition and support from employers, stakeholders and the general community so as to boost confidence and morale with the resultant effect of better healthcare delivery.

## Background

The stigmatization of Community Psychiatric Nurses (CPNs) is a highly demoralizing habit which carries far reaching negative effects on the work of CPNs in Ghana [1]. Stigma, which is referred to as a mark of shame, dishonor or discredit, is a practice associated with personal experience characterized by discrimination, rejection, denial and devaluation that results in adverse social opinions about a person or group of people [2–5]. Stigma is formed when people are wrongly seen to have an inflated negative attribute, resulting in an overall diminishing of their worth [4–7]. These negative opinions about a person or a group of people, result in increased depression, reduced hopefulness and self-esteem [2]. This deeply discredits and reduces the individual’s self-image from a whole to a soiled one with low social ranking [6]. These negative sentiments do not affect the CPN alone but also people with mental health problems who receive critical care from CPNs and their families. The trust of patients and their families in the quality of care wanes as stigma of their care givers increases. There is a pressing need for further research in order to rid the profession of these bottlenecks, so the service delivery of CPNs will improve. Many studies have been conducted into various fields of psychiatry but there is paucity in knowledge concerning the negative effects of stigma on the work of CPNs. This knowledge gap has a tendency of perpetuating and compounding stigma to the detriment of quality service delivery of CPNs to psychiatric patients in the community.

The aim of the study therefore, was to explore how stigma affected CPNs in the discharge of their duties as mental health workers in the community. This was necessary to ensure that these effects are abated in order to enable them carry out their duties with dignity.

### Literature review

Sarkar and. Punnoose detected in their study that, globally, CPNs experience a phenomenon known as associative stigma which occurs as a result of their care for mental patients; they probed 54 studies with sample sizes between 31 to 1229 participants and a total of 5871 patients. An average of 55.9% of patients experienced stigma and this level of stigma translates to CPNs by virtue of association [8].

Another study carried out in China on “Levels of stigma among community mental health staff” by [9] revealed relatively high levels of stigma in Guangzhou, amongst community mental health staff.

In Ghana it was reported that working in a mental health institution had some form of stigma associated with it. A study showed that CPNs experienced 60.6% stigma while working with other health professionals. Furthermore, 28.2% of CPNs had thought of leaving the profession to avoid stigma [10]. Likewise a study conducted by [5] in Ghana with 12 CPNs revealed that they were stigmatized because of their association with persons suffering from mental illness. Though a section of the public were genuinely concerned about the safety of these CPNs, others held a poor image, discriminated, mocked and labelled the nurses. Beyond these the CPNs experienced associative stigma because of their patients.

The nation of Ghana is situated at the south-western part of Africa, with a population of a little above thirty million (30,000,000) people. The Ghanaian economy is classified as lower-middle income. Higher education and better healthcare delivery continue to generally improve.

in spite of other setbacks. The country has in the recent past introduced the National Health Insurance Scheme (NHIS) that seeks to extend basic medical care to the general public after registration and payment of premiums [11]. There are also other private health insurance providers associated with different firms and organizations in the country [12, 13]. Despite government’s efforts to extend healthcare to the greater percentage of the people in the country, the system is ridden with so many problems that frustrate healthcare seekers in time of need [14]. The worst of it all is the shortage of certain essential medications that are supposed to be covered by the insurance scheme [15]. Patients are subsequently asked to go to specific private drug dispensaries and purchase these drugs, thus defeating the noble notion of the health insurance scheme [16].

Medication for mental conditions are not exempt from the problems stated above. Government has however made giant strides towards the wellbeing of people living with mental conditions in Ghana by the enactment of the Mental Health Bill (Act 846). This Act if properly harnessed will improve the quality of life of people with psychiatric issues. An offshoot of this law is the attachment of mental health units to all traditional general health facilities to cater for mental patients the same way any other health condition is catered for, thus making the general public accept the fact that it is also a treatable condition as all other physical health problems. Before this, only a few people with psychiatric problems received appropriate care, and that was mainly institutionalized. This service came with heavy stigmatization of patients that even rubbed off on caregivers. Despite the great investment of government and other stakeholders to improve the lot of mental patients, their efforts have produced limited results. The success of policies will also depend on the level of successful re-integration of people with psychiatric issues, but who have received treatment, into society. CPNs now play a crucial role in educating the general public on re-integration. This is being.

done but with a lot of difficulty because of stigmatization.

There is limited literature available on how stigma rubs off on CPNs and its effect on quality service delivery. This therefore necessitated a research in this area to ascertain the issues prevailing so as to provide a better understanding of the effects of stigma and determine proper solutions to it.

## Method

The pragmatist research philosophy was employed for this study. In this research philosophy, the practical results are deemed essential. This approach deals with actualities. The author engaged in retrospective reflexivity for the most part of the study.

### Research design

The study adopted the exploratory descriptive qualitative design to delve into the effect of stigma on CPNs in Accra, Ghana. An exploratory, descriptive qualitative approach makes it possible for deeper understanding of the subject being explored and also for participants of the study to put forward meaningful contributions in order to increase understanding in that area of research [17] [18]. The exploratory research design is mostly utilized when the subject under scrutiny is new or in the case when for some reason data collection for the study becomes perplexing. If a researcher has a general idea or a specific question that he/she wants to study but there is no previous knowledge or prototype with which to study it, they can employ the exploratory research design. This method was chosen because it made it possible to explore the effect of stigma on the participants’ productivity and mood. A semi-structured interview guide was used to elicit particular responses from CPNs in the Accra Metropolis.

### Setting of the study

The study took place in the Ga South, Okaikoi and Ga Central districts of the Greater Accra Region of Ghana. These 3 districts were among those with the highest number as well as the longest serving Community Psychiatric Nurses (CPNs) in the Accra Metropolis (Regional Health Directorate Community Psychiatric Units, Greater Accra).

The Ga South Municipality has a population of 310,314, in the catchment area.

Kaneshie Polyclinic is in the Okaikoi Sub-Metro, and now consists of Okaikoi North and Okaikoi South. It forms part of 13 constituencies of the Accra Metropolis of the Greater Accra Region. The district is situated at the western part of the city of Accra. The Sub-Metro has a population of three hundred and thirty thousand, one hundred and forty-four (330144).

Ga Central’s capital is Sowutuom; it is bounded North by Ga West municipality, West by Ga South municipality, and South by Accra metropolis. Ga central was carved out of Ga south and became independent on the 28th June, 2012.

### Data collection

After obtaining ethical clearance from the Noguchi Memorial Institute for Medical Research *(*NMIMR), permission was sought from the Greater Accra Regional Office of the Community Psychiatric Unit (CPU) for procedure to engage in data collection for the study.

CPNs are mandated to extend hospital care to the homes of psychiatric patients and to monitor their welfare and wellbeing as well as ensure the patients follow their drug regimes*.* The selection of CPN’s was based on the purposive sampling technique. Selected CPN’s were between the ages of 25 and 40 and must have worked for at least three years. Letters were sent to the in-charges of the CPNs of selected district health facilities together with the Ethical Clearance Certificate from Noguchi to acquire permission to engage the selected staff. The study was undertaken based on the hospitals’ protocol. The researcher booked appointments with the CPNs, established rapport with the CPNs and also sought written consent from the CPNs. Interviews were carried out with each of the participants. An interview guide was used to conduct interviews at the offices of the CPU in the various hospitals in a relaxed manner. Interviews were audiotaped and later transcribed verbatim. The researcher asked probing questions where necessary and wrote field notes at the same time or right afterwards. The interviews were conducted in English and each session lasted between forty (40) minutes to one hour twenty minutes (1 hour 20) minutes. The interviews covered demographic data and served the purpose of rapport establishment. The second section of the interview guide consisted the main guiding questions. Data collected included: public attitude towards CPNs, effects of stigma on the CPNs, problems CPNs encountered in caring for mentally ill persons in their homes and the coping mechanisms used in dealing with the stigma. Data was acquired from the perspective of the CPNs alone because they could give better explanations to the phenomena under scrutiny than the patients could.

### Data analysis

Thematic Content Analysis (TCA) was utilized for the presentation of qualitative data in a descriptive manner. Qualitative data can be acquired through interview transcripts collected from research participants or other known texts that reflect experientially on the topic of study. TCA was used because it aids researchers comprehend particular aspects of a phenomenon that participants of a study talk about repeatedly or in detail, and the means through which those aspects of a phenomenon may be linked The main aim of thematic content analysis is to analyze all aspects of the data together with the field notes. This principle involves the preparation, organizing and reporting of the results in the analysis of the transcriptions from the interviewees [19, 20].

Coding was conducted afterwards; this involved categorizing similar ideas, words, phrases and sentences and highlighting each category with a unique color. Themes and subthemes were teased from the groups formed. These were supported with verbatim quotes from the transcribed data of the participants.

In order to improve the significance of the facts gathered, data from the field notes were analyzed and added to the results. Pseudonyms were used instead of participants’ real names in order to ensure anonymity. Saturation was reached when responses to questions from the 9th to the 12th participant turned out to be similar or the same.

### Accordance

Submission was performed in accordance with the relevant guidelines and regulations of the Noguchi Memorial Institute for Medical Research. All methods were carried out in accordance with the Noguchi Memorial Institute for Medical Research relevant guidelines and regulations.

The protocol was approved by the Institutional Review Board of the Noguchi Memorial Institute for Medical Research (NMIMR-IRB).

## Results

### Demographic characteristics

The demographic data of the participants included age, gender, marital status, level of education, religion, place of work, years or duration of work and place of residence. Twelve (12) participants were successfully interviewed (1 male and 11 females). The age range of the participants was 26 years to 40 years; six (6) of them attained tertiary (bachelor’s degree) education and the other 6 had diploma in Psychiatric Nursing. Out of the 12 participants, 11 were married. All the participants were Christians. The number of years worked, ranged between 3 years and 9 years.

Three themes were developed; low productivity, depression and anger. They expressed what they had been through with health workers in other departments at the various district hospitals and also with people in their communities.

The negative attitudes and speech of colleagues in other nursing fields made the CPNs downcast and also lose confidence in their nursing abilities. These work mates did not see them as proper nurses, and therefore did not treat them properly. The ill treatment even reflected in information dissemination; vital information needed for work was often not relayed or was given out late. This definitely affected efficiency and therefore ultimately led to low productivity on the part of the affected CPNs; to aggravate this situation, logistics needed for the running of the CPN unit was lacking; their office space was also being shared with other units and this limited the effectiveness of the CPNs’ work. It was such that because patients were not given the necessary privacy of a consulting room, they were not able to fully open up about their issues due to their fear of eavesdroppers; basic things like a sphygmomanometer, blood pressure monitor among others were absent, though the CPNs had made written requests for these things, they were ignored. It gets so bad that even when CPNs write reports other colleagues feel like certain details and figures were made up. The working environment for the CPN is therefore depressive, the only way they kept going here was to apply the coping skills acquired as part of their training. Some participants wished they could switch to other better recognized departments like general nursing, midwifery, anesthesia, physician assistance and education; other CPNs admitted they sometimes reacted towards the stigma in outbursts of anger, as a defense mechanism, though most of them kept the anger within without open expression to avoid marring their already fragile image.

The following sections explore in depth the three main themes that emerged from the data.

### Low productivity

Low productivity, due to stigma, on the part of CPNs means that, all things being equal, the quality of service delivery of the average CPN to mental patients in the community fell below expectation. When the morale of workers is boosted by favorable working conditions and conducive environments they usually give off their best; when the opposite is true, low productivity is usually inevitable. Some of the participants recounted how the derision they experienced triggered an inferiority complex that prevented them from working as they were supposed to.

Participants again emphasized that health professionals in the other departments did not consider them (CPNs) as proper nurses, and as such did not relay vital information to them.

One CPN relating her experience said “*If I come to work and people, make comments that will make me unhappy how will I take care of my clients” (CPN1)?*

Another participant who had been affected by stigma in the line of duty further stated that *“If the unit is there and I am there and you don’t treat me well how do I give out my best?*

Some CPNs even believed that colleagues in other departments undermined their capacity as nurses as made evident by this participant; *“Most of them think we are not really nurses. Some staff also sees us like we don’t do anything. In some cases, they just forget about it that information they are supposed to pass on they just forget it*. *They think you are not a proper nurse. They don’t even regard us as nurses in the clinic” (CPN4).*

Others disclosed that their work was negatively affected both in the hospital setting and in their communities, because they were unable to get the necessary logistics to work with. They revealed that they felt their services were not needed because most of the time they were left out of the budget. This participant stated that, *“If I am unable to get the necessary logistics to work with, it affects our work. Our office space is used by Disease Control, Child Welfare Clinic and therefore it’s very difficult when you have clients and you want to talk to them, having privacy is a big problem. There are times a client is unable to tell you what they want, because you have a lot of other staff around. When we get aggressive clients we don’t have medications, injections for them. If we have to check patient’s blood pressure, we have to bring him all the way down to the Out Patient Department because we don’t have the blood pressure apparatus. We don’t have a sphygmomanometer; we don’t have a thermometer. We have requested for them but up till now …*” *(CPN2).* Another aggrieved caregiver claimed *“it’s like even when resources are shared they ignore us. It is here, I will say it when they are sharing resources they leave us out. It has happened several times. I don’t understand why they do that. You know when we make our reports they think we just make up figures. It does not encourage us to work” (CPN4).*

### Depressive working environment

CPNs said the only factor that kept them going on the job was applying knowledge acquired from previous trainings on how to handle traumatic situations, in order not to break down, because sometimes they could not help but feel sad, bad, unstable, hopeless due to the depressive working environment that persists. This was made evident by the CPNs when they stated the following, *“In fact, I feel very bad, aside the fact that you have the passion for the work, aside the fact that it is your job. Sometimes I feel I don’t care but it gets to us. When I am in my office or a colleague staff sees you and make certain comment you feel so bad. Where did I go wrong is it because I am a Community Psychiatric Nurse? You feel very bad, it brings your spirit down, and in fact if you don’t take care you will be depressed. I feel very bad sometimes, demoralized and sometimes angry. Feel very bad” (CPN1).*

“So sometimes it affects me emotionally I get unstable at times but sometimes I work out of it” (CPN2)*.*

“From time to time I should be frank; occasionally there is the feeling of sadness hopelessness” (CPN7)*.*

Some CPN staff wished they rather worked in other non-psychiatric care departments because of the bad treatment they received as CPNs even though they tried to deal with the situation. They felt they made a mistake to have chosen this field of nursing and were willing to make a switch when the opportunity became available; this participant reiterated that, *“No matter what we are coping. We are coping. We were all sad. I wish I was at the other department like the general or midwifery side because I think they are treated better I feel so sad and I feel so bad. Hmm it’s not pleasant at all” (CPN5).*

### Anger

Some of the participants expressed anger as one of the effects of stigma. This participant underscored that, *“It is demoralizing and sometimes I get angry*. *At times I see it to be a useless job. They should appreciate our work hmmm” (CPN1).*

The following participants highlighted that they get discouraged and angry when she blurted, “*At times I do feel very bad, at times I do regret and ask myself why I’m I doing this work? At times it discourages me and makes me angry” (CPN6).*

Speaking on self-control and anger management, as tools used to avert ruining relationships with people when provoked in the line of duty, a participant submitted that, *“because it was a church I kept quiet. When I got home I was boiling. It’s some way. If you are stigmatized and if you don’t control yourself, you will mess up. You will react, if you don’t have anger management. You will over react and then you will realize that one thing you did have marred your relationship you had with people, something you had struggled with, so it’s unpleasant for me. Sometimes I try to control my anger” (CPN7)*.

## Discussion

The study dealt with exploring the effects of stigma on CPNs in three District Hospitals in the Greater Accra Region of Ghana. Even though there are adverse effects of stigma on CPNs in Ghana, little literature exists about aspects of Community Psychiatric Nursing, such as low productivity, depression, and anger.

Most of the participants of the study recounted how stigmatization had affected their work both in the hospital setting and in their communities, [10]. They said that some community mental health workers in Ghana have considered leaving the mental health profession because they have been harmfully impacted by the stigma in mental health and had to leave the profession entirely to other professions like general nursing, midwifery, anesthesia, physician assistantship and education.

Others also said it was terrible to the extent that they were unable to get the necessary logistics for their work [21]. said it was not easy for the CPNs because of the difficulty in locating the homes of patients due to poor home addresses and unavailability of transportation, limited logistical support and irregular supply of medication. A lack of these facilities deter many young potential nurses from branching into community psychiatric nursing. If no one plans to further as a psychiatric mental health nurse, there will be shortage of staff thereby reducing service delivery and productivity [22].

Meanwhile most of the participants said they could not help but feel sad, bad, unstable, hopeless and depressed because of the way they are treated by the public. Caregivers’ hopelessness was determined or predicted by patients’ state [23] and that patients’ depression is correlated to that of the caregiver [24]. The caregivers of patients may be just as likely to experience loneliness or depression. Those who worry more about public stigma associated with mental illness, suffer depression [25]. In a related study conducted in Germany, [26] anticipated stigma could result in reduced quality of life and depression [27]; discovered specifically that internalized stigma was linked with depression. In addition there was anger as one of the effects of stigma on CPNs because some of the participants said they could not help but get angry due to the way they are treated [28]. The fact that the CPN profession is stigmatized means, the value or worth of that field of endeavor is waned and will keep depreciating in the public eye. This will not attract new entrants into the field, which will ultimately lead to a natural death of the profession, unless something drastic is instituted to pull out the reputation of the CPN and that of their work from the mud of stigma. That said, the group that stands to lose most will be people with mental illnesses [29], especially those in the communities, because with a crumbling CPN profession there will be little or no care for the many psychiatric conditions prevailing in our society, this will in turn lead to multiple relapses and have a final effect of making the community lose precious human resource, necessary for general societal development, to mental illness.

## Conclusion

Low productivity, depression and anger were some of the effects of stigmatization on CPNs. These were based on participants’ expressions. It is therefore necessary that all heads of departments of district hospitals put measures in place to deal with stigmatization of CPNs in their districts and communities, this is because the primary role of the CPN is to extend clinical care to the homes of patients who have been discharged, ensuring the treatment regimen is followed in order to ensure full recovery of patients and also to assist in the re-integration of patients into society. That said, a demoralized CPN will not have the capacity to fully execute the job, and coupled with the fact that many CPNs are seeking ways to switch to other non-psychiatric fields, the group that stands to lose most are psychiatric patients. This among other things will cause the percentage of patient relapses to rise. CPNs therefore need all the respect and support to make their work easier and to also perform their work effectively and help reduce relapses of persons with mental illnesses. Furthermore, CPNs will need more support from the mental health authority as well as the media to enable them perform their work and also boost their public image, making it void of stigma.

## Data Availability

The datasets generated and/or analyzed during the current study are not publicly available due to limitations of ethical approval involving the community psychiatric nurses data and anonymity but are available from the corresponding author on reasonable request.

## Abbreviations

CPNs: Community Psychiatric Nurses
NHIS: National Health Insurance Scheme
NMIMR: Noguchi Memorial Institute for Medical Research
CPU: Community Psychiatric Unit
CPN: Community Psychiatric Nurse
MPhil: Master of Philosophy
